# Co-Designing a Virtual Reality Application to Enhance Reminiscence Therapy for Persons with Dementia

**DOI:** 10.1093/geroni/igab046.3575

**Published:** 2021-12-17

**Authors:** Winnie Sun, Alvaro Quevedo

**Affiliations:** Ontario Tech University, Oshawa, Ontario, Canada

## Abstract

Reminiscence therapy for persons with dementia is often being conducted by employing analog media including pictures and videos organized and presented by a caregiver. However, such media lacks the immersive experience to support patient engagement and successful recollection of reminiscence events. Recently, Virtual Reality (VR) is gaining momentum as a potential technological tool to support dementia care due to its increased immersion, presence, and embodiment. Haptic artifacts can be used to enrich reminiscence therapy as part of the multi-sensory stimuli to increase immersion and patient engagement, as well as improving social connectedness and cognitive health. The purpose of this project is to explore the use of VR application to advance reminiscence therapy for persons with dementia. We have prototyped an immersive and non-immersive VR framework that allows caregivers to deliver reminiscence therapy for persons with dementia with varying stages in their disease progression. These reminiscence therapy sessions are built by employing a narrative storyboard and content management through a series of co-designing sessions with content experts at the Geriatric Dementia Unit in Ontario, Canada. A caregiver-led VR framework will be adopted to enable the caregiver to guide the persons with dementia to safely navigate through the interactive VR environment, while allowing the patients to engage with the interactive VR elements using a point-and-pinch gesture approach. We anticipate that the VR experiences hold the potential for improving the interactions between persons with dementia and caregivers, as well as enhancing the reminiscence experiences to promote the maximal therapeutic benefit of patient’s recovery.

